# Suicidality in post-traumatic stress disorder (PTSD) and complex PTSD (CPTSD)

**DOI:** 10.1192/j.eurpsy.2021.390

**Published:** 2021-08-13

**Authors:** L. Longo, T. Jannini, M. Merlo, V. Cecora, M. Gagliano, B. D’Imperia, A. Daverio, L. Monaco, R. Rossi, C. Niolu, A. Siracusano, G. Di Lorenzo

**Affiliations:** Department Of Systems Medicine, University of Rome Tor Vergata, Rome, Italy

**Keywords:** cPTSD, ptsd, Suicidality, Hopelessness

## Abstract

**Introduction:**

International Classification of Diseases 11th Revision (ICD-11) has inserted complex post-traumatic stress disorder (cPTSD) as a clinically distinct disorder, different from PTSD. The diagnosis of cPTSD has the same requirements for the one of PTSD, in addition to disturbances of self-organization (DSO – e.g., disturbances in relationships, affect dysregulation, and negative self-concept).

**Objectives:**

This study aimed to explore suicidality in PTSD and cPTSD. We examined also the association between clinical dimensions of hopelessness (feelings, loss of motivation, future expectations) and other symptomatologic variables.

**Methods:**

The sample, recruited at the Fondazione Policlinico Tor Vergata, Rome, Italy, consisted of 189 subjects, 132 diagnosed with PTSD, and 57 with cPTSD, according to the ICD-11 criteria. Participants underwent the following clinical assessments: Clinician-Administered PTSD Scale (CAPS), Impact of Event Scale-Revised (IES), Beck Depression Inventory (BDI), Symptom Checklist-90-Revised (SCL-90), Dissociative Experience Scale (DES), Beck Hopelessness Scale (BHS).

**Results:**

cPTSD showed significantly higher BHS-total (p = 0.01) and BHS-loss of motivation subscale (p <0.001) scores than PTSD. Besides, cPTSD showed significantly higher scores in all clinical variables except for the IES-intrusive subscale. By controlling for the confounding factor “depression”, suicidality in cPTSD (and in particular the BHS-total) appears to be correlated with IES-total score (p = 0.042) and with DES-Absorption (p = 0.02). Differently, no such correlations are found in PTSD.

**Conclusions:**

Our study shows significant symptomatologic differences between PTSD and cPTSD, including suicidality. Indeed, suicidality in cPTSD appears to be correlated with the “loss of motivation” dimension, which fits well within the ICD-11 criteria of DSO.

**Disclosure:**

No significant relationships.

